# A Single Amino-Acid Substitution Allows Endo-Polygalacturonase of *Fusarium verticillioides* to Acquire Recognition by PGIP2 from *Phaseolus vulgaris*

**DOI:** 10.1371/journal.pone.0080610

**Published:** 2013-11-19

**Authors:** Manuel Benedetti, Federico Andreani, Claudia Leggio, Luciano Galantini, Adele Di Matteo, Nicolae Viorel Pavel, Giulia De Lorenzo, Felice Cervone, Luca Federici, Francesca Sicilia

**Affiliations:** 1 Dipartimento di Biologia e Biotecnologie “Charles Darwin”, Sapienza Università di Roma, Roma, Italy; 2 Dipartimento di Chimica, Sapienza Università di Roma, Roma, Italy; 3 Istituto di Biologia e Patologia Molecolari, Consiglio Nazionale delle Ricerche, Roma, Italy; 4 Dipartimento di Scienze Sperimentali e Cliniche and Centro Scienze dell’Invecchiamento, Università di Chieti-Pescara “G. d’ Annunzio”, Chieti, Italy; Iowa State University, United States of America

## Abstract

Polygalacturonases (PGs) are secreted by phytopathogenic fungi to degrade the plant cell wall homogalacturonan during plant infection. To counteract Pgs, plants have evolved polygalacturonase-inhibiting proteins (PGIPs) that slow down fungal infection and defend cell wall integrity. PGIPs favour the accumulation of oligogalacturonides, which are homogalacturonan fragments that act as endogenous elicitors of plant defence responses. We have previously shown that PGIP2 from *Phaseolus vulgaris* (PvPGIP2) forms a complex with PG from *Fusarium phyllophilum* (FpPG), hindering the enzyme active site cleft from substrate. Here we analyse by small angle X-ray scattering (SAXS) the interaction between PvPGIP2 and a PG from *Colletotrichum lupini* (CluPG1). We show a different shape of the PG-PGIP complex, which allows substrate entry and provides a structural explanation for the different inhibition kinetics exhibited by PvPGIP2 towards the two isoenzymes. The analysis of SAXS structures allowed us to investigate the basis of the inability of PG from *Fusarium verticilloides* (FvPG) to be inhibited by PvPGIP2 or by any other known PGIP. FvPG is 92.5% identical to FpPG, and we show here, by both loss- and gain-of-function mutations, that a single amino acid site acts as a switch for FvPG recognition by PvPGIP2.

## Introduction

Pectin is the outer component of the plant cell wall and, therefore, is among the first structures to be challenged during pathogen invasion or wounding [1]. To gain access to the plant tissue, pathogens secrete cell wall-degrading enzymes (CDWEs), including endo-polygalacturonases (PGs) that cleave the α-1,4 linkages between D-galacturonic acid residues in the homogalacturonan and cause cell separation and maceration of the host tissue [2]. PGs are virulence factors of many phytopathogenic fungi such as *Botrytis cinerea* [3], *Sclerotinia sclerotiorum* [4], *Claviceps purpurea* [5] and bacteria such as *Agrobacterium tumefaciens* [6] and *Ralstonia solanacearum* [7]. To counteract PG activity, plants have evolved gene families encoding PG-inhibiting proteins known as PGIPs [8,9]. PGIPs inhibit the activity of PGs and favor the accumulation of oligogalacturonides (OGs), oligomers of galacturonic acid with a degree of polymerization ranging from 9 to 15 [10-12]. OGs are among the best-characterized plant damage-associated molecular patterns (DAMPs) [13-16] and are specifically recognized by receptor proteins belonging to the wall-associated kinase family [17]. The importance of PGIPs in defense is well documented by studies showing that plants where the expression of PGIPs is partly silenced are more susceptible to fungal infection [18] and, conversely, that transgenic plants overexpressing the inhibitor are more resistant to fungi [19-23]. The PG-PGIP interaction is paradigmatic for studying the key recognition events that underlie plant immunity [24]. PGIP belongs to the extracellular Leucine-Rich Repeat (eLRR) family of proteins [25], like the majority of proteins encoded by the so-called plant resistance (R) genes [12,26]. Distinct PGIP isoforms that display different specificity of recognition for PGs are produced by plants [25,27]. The isoform 2 of *Phaseolus vulgaris* (PvPGIP2) is the best characterized inhibitor and has the strongest inhibitory activity against most of the tested PG from different pathogens [23,28]. Genes encoding PGIPs are under selection pressure for diversification and a number of hot spots for the interaction with PGs have been identified in the LRR concave surface of the inhibitor [27]. Furthermore it has been shown that a few PGIP residues, sometimes only one, are critical for a stable PG-PGIP interaction [27,29]. 

 A low resolution structure of the complex formed by PvPGIP2 and PG from *Fusarium phyllophilum* (FpPG) was recently solved by Small-Angle X-ray Scattering (SAXS) [30]. This allowed to pinpoint the residues involved in the FpPG-PvPGIP2 interaction and explained the competitive inhibition played by the inhibitor on FpPG. However, PvPGIP2 inhibits different PGs with different inhibition mechanisms [31-33]. For instance, the PGs from *Colletotricum lupini* (CluPG1) and from *Aspergillus niger* (AnPGII) are non-competitively inhibited by PvPGIP2 [31,33]. The hypothesis that PvPGIP2 might recognize different PGs by forming complexes of different shape was proposed [9]. Here we provide experimental evidence that this is indeed the case. We investigate, by SAXS analysis, the complex formed by CluPG1 and PvPGIP2 and show that, unlike in the FpPG-PvPGIP2 complex, the enzyme active site cleft is still accessible to substrate entry. The structural data on these PG-PGIP complexes allowed us to identify crucial residues that explain why PG from *Fusarium verticilloides* (FvPG) is not inhibited by any known PGIP, including PvPGIP2. Indeed we show that a single amino acid substitution, with respect to FpPG, allows this enzyme to escape PvPGIP2 recognition. The data reported here suggest possible strategies for the genetic manipulation or selection of *PGIP* genes with new recognition capabilities. 

## Materials and Methods

### CluPG1 and FvPG Expression and Purification

The Polygalacturonase 1 of *Colletotrichum lupini* (CluPG1) strain SHK78813, was purified from the fungal culture medium as already described [33]. 

The polygalacturonase of *Fusarium phyllophilum* (FpPG) was expressed and purified as previously reported [30].

The cDNA encoding the polygalacturonase of *Fusarium verticillioides* (FvPG) strain 62264 was cloned in pGAPZαA (Invitrogen) using the EcoRI and XbaI restriction sites introduced by using the primers FvPGEcoFw and FvPGXbaRv (Table S1). The construct, generated in frame with the signal sequence for secretion of the yeast (*Saccharomyces cerevisiae*) α factor, was amplified by transforming *Escherichia coli* TOP10F competent cells (Invitrogen). pGAPZαA-FvPG was extracted from the cells using a plasmid mini prep kit (Qiagen) and analyzed by digestion with EcoRI and XbaI restriction enzymes, followed by 1% (w/v) agarose gel analysis. The plasmid DNA was linearized with AvrII restriction enzyme and used to transform *Pichia pastoris* X33 cells (Invitrogen) by electroporation. The selection of the zeocin-resistant *P. pastoris* transformants was carried out according to the manufacturer’s instruction (Invitrogen). The medium used for growth of *P. pastoris* contained 1% (w/v) yeast extract, 1% (w/v) tryptone and 2% (w/v) glucose. The filtrate obtained from 3-days-old culture was concentrated using a Vivaflow 200 (Sartorius Stedim) and dialyzed against 20 mM Sodium Acetate (NaOAc) pH 4.0. The dialyzed sample was loaded on a diethylaminoethyl cellulose resin (DE52, Whatman) pre-equilibrated with 20 mM NaOAc pH 4.0. The flow through was then loaded on a HiTrap SP-Sepharose column (GE Healthcare), pre-equilibrated with 20 mM NaOAc pH 4.0. Eluition was carried out using a linear gradient of NaCl (from 0 to 1 M) in the same buffer. The fractions that showed the highest PG activity were pooled and dialyzed against 20 mM NaOAc pH 4.0. Subsequently, ammonium sulfate was added to the dialyzed proteins to reach 2 M final concentration and the sample was loaded on a HiTrap Phenyl-Sepharose column (GE Healthcare) pre-equilibrated with 20 mM NaOAc pH 4.0 and ammonium sulphate 2 M. Elution was performed by decreasing the concentration of ammonium sulphate (from 2 M to 0 M in 10 min) in 20 mM NaOAc pH 4.6. Purified fractions as determined by SDS-PAGE were pooled.

### PvPGIP2 Expression and Purification

The PG-inhibiting protein 2 of *Phaseolus vulgaris* pv Pinto (PvPGIP2) was cloned in pGAPZαA (Invitrogen), expressed in *Pichia pastoris* X33 (Invitrogen) and purified as previously described [30]. 

### CluPG1-PvPGIP2 and FvPG-PvPGIP2 Chemical Cross-Linking

The crosslinking reaction between PvPGIP2 and CluPG1 or FvPG was performed as previously described [30]. Seventy micrograms of PvPGIP2 were cross-linked to 78 µg of CluPG1 (molar ratio 1:1) in 200 µL of a solution containing 50 mM NaOAc pH 4.6 supplied with fresh 1% methanol-free formaldehyde (Thermo-Fisher Scientific). The reaction was incubated at 28°C for 16 h and finally concentrated to 2 µg of total proteins/mL. The single proteins used as negative control were cross-linked using the same reaction conditions. Cross-linked proteins and negative controls were analyzed by SDS-PAGE.

### Site Directed Mutagenesis

*FpPG, FvPG* and *PvPGIP2* genes cloned in the pGAPZαA vector, were used as templates for site directed mutagenesis using the QuickChange® II Site-Directed Mutagenesis Kit (Agilent) according to the manufacturer’s instructions. The forward and reverse primers used to introduce the mutations in the corresponding positions of *PvPGIP2* (Q224K, Q224E), *FpPG* (L303E, K310T, S363K, K116E, S120N, N121K, S122D, Q124P and S120N-N121K-S122D), *FvPG* (T274A and L303E) are listed in Table S1. The mutated genes were sequenced to confirm the presence of the desired mutations and subsequently used to transform *P. pastoris*, as heterologous expression system as described above. 

PGIP2.Q224K and PGIP2.Q224E were purified using the same procedures described for the native PvPGIP2 [30].

### Agar diffusion assay

The capability of wild-type and mutant PvPGIPs to inhibit the activity of the native and variant forms of FpPG, and FvPG was measured by the agar diffusion assay as previously described [20]. PG activity was expressed as agarose diffusion units and one unit was defined as the amount of enzyme that produced a halo of 0.5 cm radius (external to the inoculation well) after 16 h at 30°C. The agarose inhibition unit was defined as the amount of PvPGIP2 causing 50% inhibition of 1 agarose diffusion unit at pH 4.7.

### SAXS data acquisition and analysis

SAXS has become a powerful tool to decipher three-dimensional low resolution structures [34-36]. Due to the progress of ab initio and rigid body modeling [37-39] the three-dimensional shapes, consistent with the pair distribution functions, can be assessed for monomeric and multimeric state of proteins as well as protein complexes [40,41]. In addition SAXS can provide structural information about self-aggregated systems, multidomain and intrinsically unfolded proteins [42-46], mechanisms of denaturation and unfolding [43,47], binding of small molecules and their stabilizing role in denaturating conditions [48,49], and molecular mechanisms of ligand release [50].

Prior to SAXS analysis, the cross-linked CluPG1-PvPGIP2 sample was concentrated up to 22 μM. SAXS measurements were carried at 25.0°C in a quartz capillary of 1 mm diameter by using a Kratky Compact camera (Anton Paar), with a slit collimation system and equipped with a NaI scintillation counter. The nickel-filtered copper Kα radiation (λ = 1.5418 Å) was used and scattering curves were recorded within the range of 0.01 ≤ q ≤ 0.4 Å^-1^ (q = 4πsinθ/λ, where 2θ is the scattering angle). The intensity of the primary beam was measured by employing the moving slit method. The collimated scattering intensities were put on an absolute scale, corrected for the solvent and the capillary contributions, and expressed in electron units eu (electrons^2^ Å^-3^) per centimetre primary-beam length [51,52]. One eu corresponds to 7.94056 x10^-2^ cm^-1^ in terms of total scattering cross section of a particles ensemble [53]. Spectra were interpreted by the Indirect Fourier transform method as implemented in the ITP program [54]. For very dilute samples (no particle interactions) the scattered intensity, I(q), can be related to the pair distribution function p(r) of the single scattering particle according to the equation:

$I(q)=\int_{0}^{\infty }p(r)\frac{sin(qr)}{qr}dr$

On the basis of this equation, the ITP extracts of the p(r) function from the desmeared scattering pattern. The p(r) function is strongly dependent on the shape and size of the scattering particles and vanishes at the maximum particle size D_max_. Furthermore, it permits the determination of the electronic radius of gyration R_g_[54]. The obtained values are more accurate than those derived from the Guinier approximation [55]. Absolute intensity values reliability and instrumental set up correcteness were checked determining the mass, the gyration radius and the p(r) function of defatted Human Serum Albumin in solution at pH 7.4 [56]. The resolution limit allows analysing particles with a maximum dimension of 310 Å. Each SAXS measurement was obtained averaging three consecutive runs. The superimposition of the patterns allowed to exclude the formation of oligomers or a damage of the sample due to the X-ray radiation exposure. The presence of oligomers can be also detected by examining the Guinier plot (lnI(q) vs q^2^) and the p(r) function [57]. Only in the case of the FvPG-PGIP2 complex, aggregation was detected after 24 hours.

Shape reconstruction was performed by both *ab initio* methods and rigid body modelling, which allow to obtain three dimensional envelopes in solution with a resolution less than 15 Å. The *ab initio* method as employed in the GA_STRUCT program was used to analyse our data [58]. Starting from an aggregate of spheres, related to the expected volume and the D_max_ of the scattering particle, the p(r) was calculated by means of a Monte Carlo method. A fitting parameter was determined from the calculated (Fourier Transform) I(q) and the experimental one. A linear minimization was performed using a genetic algorithm that improves 50 models by means of mating, mutation and extinction operations. At the end, all models are docked and a consensus envelope is constructed from the 70% of models with the highest total docking score. Analysis of the SAXS intensity profiles was also based on rigid body modelling, taking advantage of the available crystal structures of the PvPGIP2, FpPG and ClPG1 proteins [25,33,59]. A simulated annealing protocol within the program SASREF [60] was employed to construct an interconnected ensemble of subunits without steric clashes, while minimizing the discrepancy between the experimental scattering data and the curves calculated from the appropriate subunits assemblies. In this procedure, the scattering patterns I(q) of each protein were calculated with the program CRYSOL [61], starting from the high resolution crystal structures. Six different SASREF reconstructions were analyzed by the DAMAVER program [62] and the various calculated complex models were superimposed by the SUPCOMB program [61]. The program gives a normalized spatial discrepancy (NSD) value related to the overlap goodness and is used as a parameter to determine the difference between two three-dimensional objects. From the cross-correlation NSD table a mean value over all pairs <NSD> and dispersion Δ(NSD) were calculated in order to discard possible outliers with NSDk values exceeding <NSD> + 2Δ(NSD). All models obtained by the SASREF analysis and subsequently subjected to the DAMAVER program, passed this test. This represents a further confirmation of the strict similarity among the SASREF structures, which were extracted without any bias.

## Results

### SAXS Analysis of the CluPG1-PvPGIP2 complex

**Figure 1:**
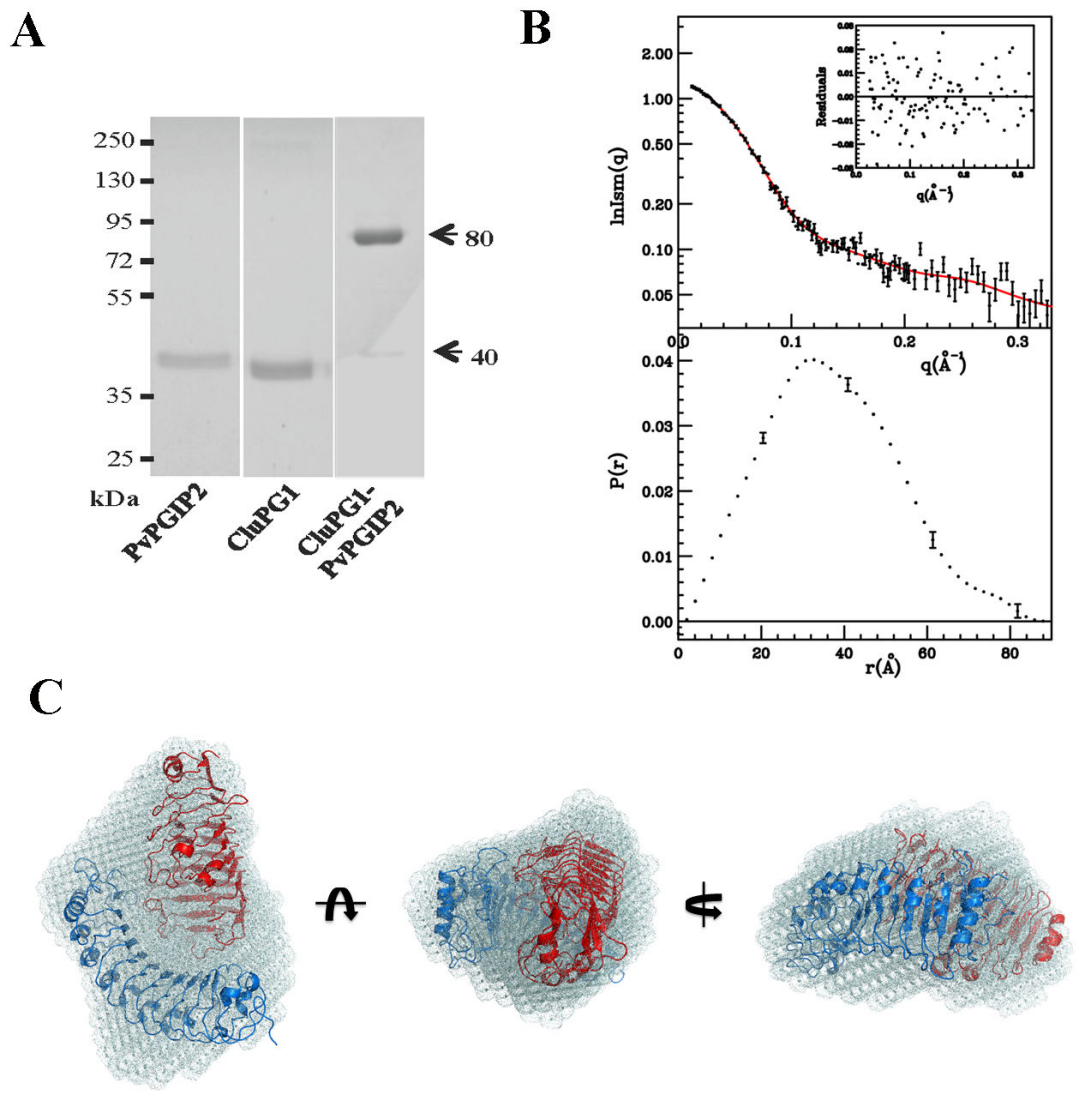
Analyses on the cross-linked complex formed by CluPG1 and PvPGIP2. A, SDS-PAGE analysis on the CluPG1-PvPGIP2 cross-linked complex. B, SAXS analysis on CluPG1-PvPGIP2 cross-linked complex: experimental scattered intensities (dots) and the calculated ones (line) are shown in the top panel. The residuals are reported in the inset and the corresponding p(r) function is shown in the bottom panel. For clarity only a few error bars are shown. C, Three-dimensional structure of the cross-linked complex between CluPG1 (in red) and PvPGIP2 (in blue) obtained with the SASREF program superimposed to the consensus envelope calculated by the GA_STRUCT program. Three orthogonal viewpoints are represented.

**Figure 2:**
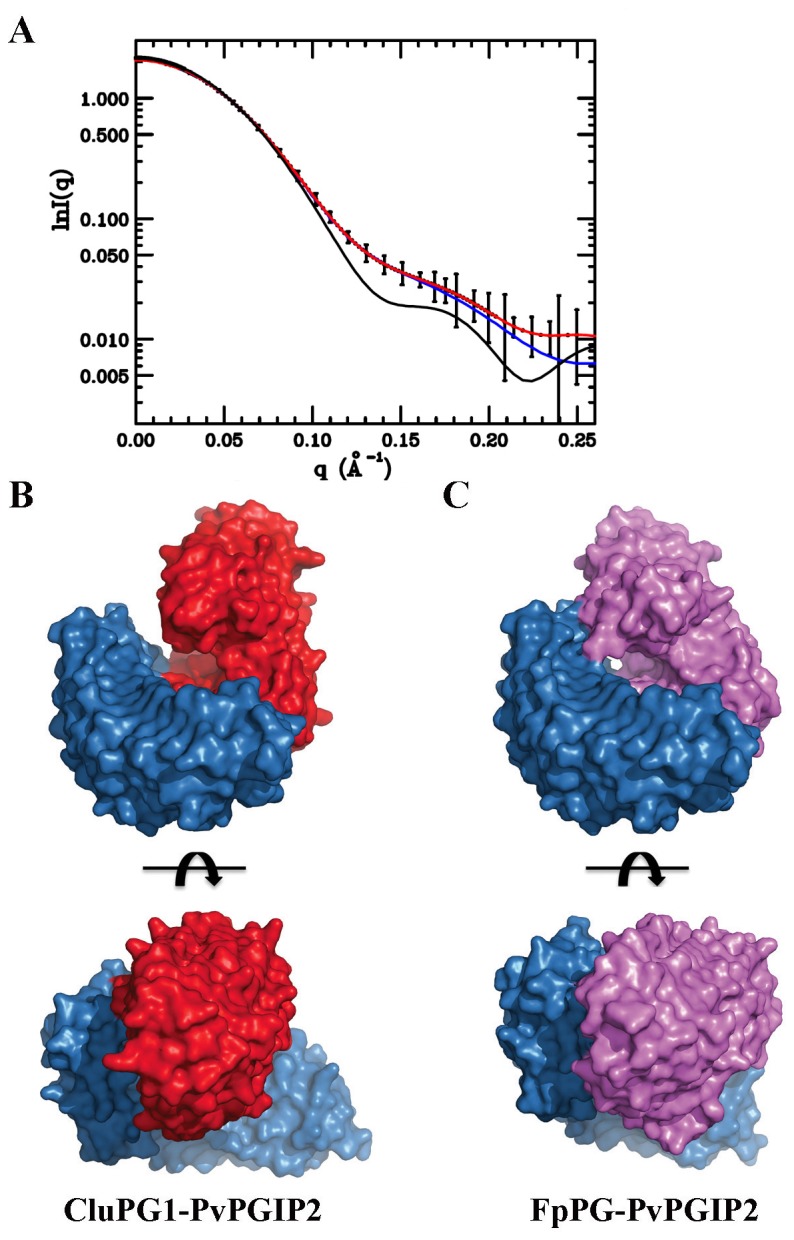
Comparative analysis between the CluPG1-PvPGIP2 and FpPG-PvPGIP2 complexes. A, The CluPG1-PvPGIP2 (red) and FpPG-PvPGIP2 scattering patterns (black) are reported together with the profile by the SASREF program for the CluPG1-PvPGIP2 complex (blue), for comparison. B, Surface representation of the complex formed by CluPG1 (in red) and PvPGIP2 (in blue). C, Surface representation of the complex formed by FpPG (in magenta) and PvPGIP2 (in blue). PvPGIP2 contacts both edges of the active site cleft in FpPG while only one edge is contacted in CluPG1. Two orthogonal viewpoints are reported.

We investigated by SAXS analysis the interaction between CluPG1 and FvPGIP2, characterised by a non-competitive inhibition mechanism [33]. The analysis was performed at pH and ionic strength conditions that mimic the plant cell wall environment at the early stage of fungal infection. We performed SAXS measurements using a PG-PGIP complex stabilized through chemical cross-linking, in order to improve the data resolution and overcome the continuous association and dissociation of the two proteins during the time course of the experiments [30]. The cross-linking reaction produces a heterodimeric complex (approximately 80 kDa) with a yield higher than 90%, as indicated by the low amount of free proteins observed (Figure 1A). The experimental and calculated scattering intensities and the corresponding p(r) function are reported in Figure 1B. A gyration radius Rg = 28.5 ± 0.4 Å and a maximum particle size Dmax = 84 ± 2 Å were obtained. Parameters obtained from SAXS analysis are reported in Table S2. To reconstruct the shape of the CluPG1-PvPGIP2 complex, the scattering pattern was analyzed by the GA_STRUCT [58] and the SASREF [60] programs which use *ab initio* and rigid body docking protocols, respectively. To exclude the possibility that multiple and divergent solutions might exist that equally fit the experimental data, the SASREF protocol was repeated six times. The mean agreement index value for the superimpositions of five structures with the sixth taken as reference is 1.23 ± 0.06. A check with the experimental data gives a mean agreement index value between observed and calculated intensities of < χ> = 0.49 ± 0.04 that means a SD of less than 8%. The results obtained with the two different methods were compared using the DAMAVER program [62]. The six superimposed structures showed a good degree of superimposition (NSD = 0.69) with the corresponding calculated consensus envelope. The model obtained for the CluPG1-PvPGIP2 complex superimposed to the consensus envelope is shown in Figure 1C. The two models obtained by different methods are consistent with each other. PvPGIP2 engages the CluPG1 β-helix with its concave surface and interacts mainly with the C-terminal edge of the enzyme active site cleft, leaving unhampered substrate access. Thus, the shape of the CluPG1-PvPGIP2 complex is different from that of the FpPG-PvPGIP2, previously solved by SAXS analysis [30]. The scattered intensities for both pairs are shown in Figure 2A and their remarkable differences are reflected in the calculated models of enzyme-inhibitor complexes reported in Figure 2B and 2C. PvPGIP2 contacts CluPG1 only at the C-terminal edge of the active site cleft (Figure 2B), while both edges of the FpPG active site cleft are engaged by the inhibitor in the FpPG-PvPGIP2 complex (Figure 2C). In the CluPG1-PvPGIP2 complex the active site is still accessible to substrate, potentially allowing the formation of a ternary complex with the substrate while, in the FpPG-PvPGIP2 complex, the active site access is completely covered by the inhibitor. Indeed, PvPGIP2 inhibits CluPG1 through a non-competitive mechanism [33], while FpPG is inhibited competitively [59]. Hence, these data are in agreement with the inhibition kinetics observed. 

#### Structure-guided mutations of FpPG highlight the role of a partially conserved surface within PGs

**Figure 3:**
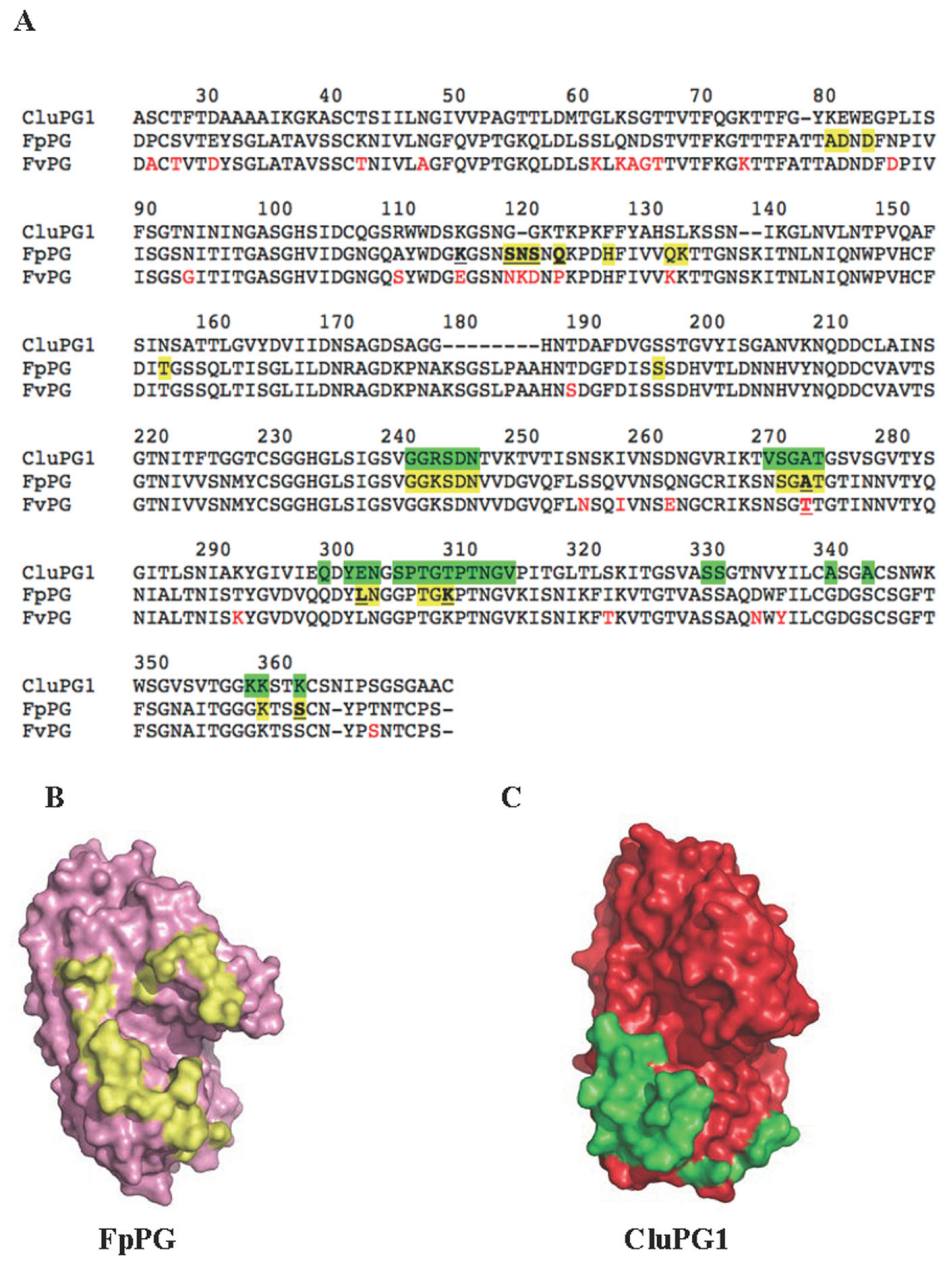
Comparative analysis of the PG amino acids contacted by PvPGIP2. A, Alignment of CluPG1, FpPG and FvPG amino acid sequences. The amino acids belonging to CluPG1 and to FpPG that are contacted by PvPGIP2 are highlighted in green and yellow, respectively. The amino acids belonging to FvPG that differ from the corresponding residues of FpPG are shown in red. Residues subjected to site-directed mutagenesis are underlined. B, Surface representation of FpPG; the amino acids contacted by PvPGIP2 are reported in yellow. C, Surface representation of CluPG1; the amino acids contacted by PvPGIP2 are reported in green.

The structures of CluPG1-PvPGIP2 (this work) and FpPG-PvPGIP2 complexes [30] were analysed by the ContPro software (http://procarb.org/contpro) [63] to identify residues of PGs located at a distance smaller than 6 Å from the inhibitor (Figure 3A). The interacting residues of FpPG are located at both the N-terminal and C-terminal edges of the active site cleft and are only partially conserved (Figures 3A and 3B). Instead the interacting residues of CluPG1 are located only at the C-terminal edge of the active site (Figures 3A and 3C). Among the interacting residues of CluPG1, three (E290, T297 and K350) are replaced in FpPG by non-conservative substitutions (L303, K310 and S363, respectively) (Figure 3A). We mutated these FpPG residues into the corresponding ones of CluPG1, generating three FpPG mutants (FpPG.L303E, FpPG.K310T and FpPG.S363K). The inhibitory capability of PvPGIP2 against these mutants was tested by agarose diffusion assay and no difference with respect to the wild type enzyme were observed, confirming that both enzymes are contacted by PvPGIP2 in this area (Table I). 

**Table I:** Inhibitory activities of native and site-directed mutants of PvPGIP2 (Q224K and Q224E) versus CluPG1 and the wild type (wt) and mutated forms of FpPG (L303E, K310T and S363K).

		**PvPGIP2**	
	wt	Q224K	Q224E
**CluPG1**	2.5	2.5	1.1
**FpPG**	2	∞*	2
**FpPG L303E**	2	20*	2
**FpPG K310T**	2	∞*	2
**FpPG S263K**	2	∞*	2

Amount of wt and mutated forms of PGIP causing 50% inhibition of one agarose diffusion unit of the indicated PGs are shown. Asterisks indicate statistically significant differences, according to Student's t test (* P < 0.003). The symbol ∞ indicates > 1500 ng.

Residue Q224 of PvPGIP2 was previously shown to be crucial for the interaction with FpPG [27,29]. This is confirmed here by the observation that the PvPGIP2.Q224K variant is unable to inhibit FpPG; on the contrary, PvPGIP2.Q224K maintains unaltered the inhibitory capability towards CluPG1 (Table I). PvPGIP2.Q224K was also tested against the FpPG mutants. Interestingly, PvPGIP2.Q224K inhibited the enzymatic activity of the mutant FpPG.L303E, albeit to a lesser extent with respect to the wild type PvPGIP2, and did not inhibit that of the other mutants (Table I). SAXS data suggest that PvPGIP2.Q224 is not in contact with FpPG.L303 [30] and indeed we show that the PvPGIP2.Q224E variant [64] is still able to inhibit both FpPG and FpPG.L303E (Table I). One possible explanation for these findings is that, while the C-terminal edge of active site clefts of both FpPG and CluPG1 is contacted by PvPGIP2, the interaction in this area is stronger with CluPG1 due to the presence of a glutammate at position 303, which however does not make contact with residue Q224 of PvPGIP2. This residue is instead crucial for recognition of only FpPG, at a site different from L303, as indicated from SAXS analysis, and this recognition is abolished by the Q224K mutation. Mutation of FpPG L303 into the CluPG1 glutammate may reinforce the binding energy enough to compensate the effect of the PvPGIP2 Q224K mutation, thus allowing the formation of a PvPGIP2.K224-FpPG.E303 complex. Collectively these data suggest that the area surrounding position 303 in PGs is crucial to determine whether the enzymes are inhibited by PGIPs.

### A gain of function mutation enables FvPG to be recognized by PvPGIP2

**Figure 4:**
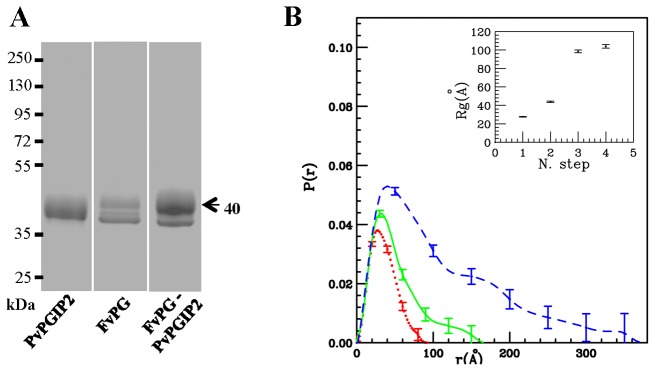
Analysis of the complex formed by FvPG and PvPGIP2. A, SDS-PAGE analysis of the FvPG-PvPGIP2 cross-linked complex. The single proteins are also reported. B, Calculated pair distribution functions of the first three SAXS patterns collected for the FvPG-PvPGIP2 complex; red dot-line, green continuous-line and blue dash-line correspond to three consecutive data sets (interval times about 20 h), respectively. In the inset, the Rg values of four consecutive data sets are shown.

PG from *Fusarium verticilloides* strain 62264 (FvPG) is 92.5% identical to FpPG with only 30 amino acid variations over 373 residues in the mature enzymes (Figure 3A). Despite this high conservation, PvPGIP2 is unable to inhibit FvPG [65-67]. This lack of inhibition may be either due to a lack of interaction or to the formation of a complex in which PG enzymatic activity is not affected. Cross-linking experiments under the same conditions as those used for the PvPGIP2-CluPG1 (Figure 1A) pair, showed the absence of a band at the expected molecular weight for the heterodimeric complex (Figure 4A). This was confirmed by SAXS analysis on a non-cross-linked PvPGIP2-FvPG solution: the evolution of the p(r) functions and Rg values (as a function of the time course of the experiment with steps of 20 hours), illustrated in Figure 4B, suggests that no stable complex between the two proteins is formed, while a progressive aggregation of the sample starts after about 24 hours. We concluded that PvPGIP2 is unable to inhibit FvPG activity because it cannot interact with this enzyme.

In order to identify the residues of FvPG responsible for the lack of recognition by PvPGIP2, we analysed the amino acid variations between FpPG and FvPG in the areas that are recognized by the inhibitor, as revealed by the SAXS data. Main variations are located at the N-terminal edge of the active site cleft (FpPG residues S120, N121, S122 and Q124) while only one variation (A274) is located in the interacting area at the C-terminal edge (Figure 3A). We mutated each of these FpPG residues into the corresponding ones of FvPG, in a loss-of-function approach, and generated the single mutants FpPG.S120N, FpPG.N121K, FpPG.S122D, FpPG.Q124P and FpPG.A274T. In addition, we generated the triple mutant FpPG.S120N-N121K-S122D, and the variant FpPG.K116E, as a negative control. The residue at position 116 is replaced non-conservatively and is located in close proximity to the interaction area but does not interact with PvPGIP2, according to SAXS data [30]. To rule out the possibility that the mutations cause a variation of the PG expression levels and a possible alteration of the specific activity, western blotting analysis of amounts of mutated PGs producing an equal activity on the agar diffusion assay was performed (Figure S1). No significant effect of the mutations on the FpPG enzymatic activity was observed.

**Figure 5:**
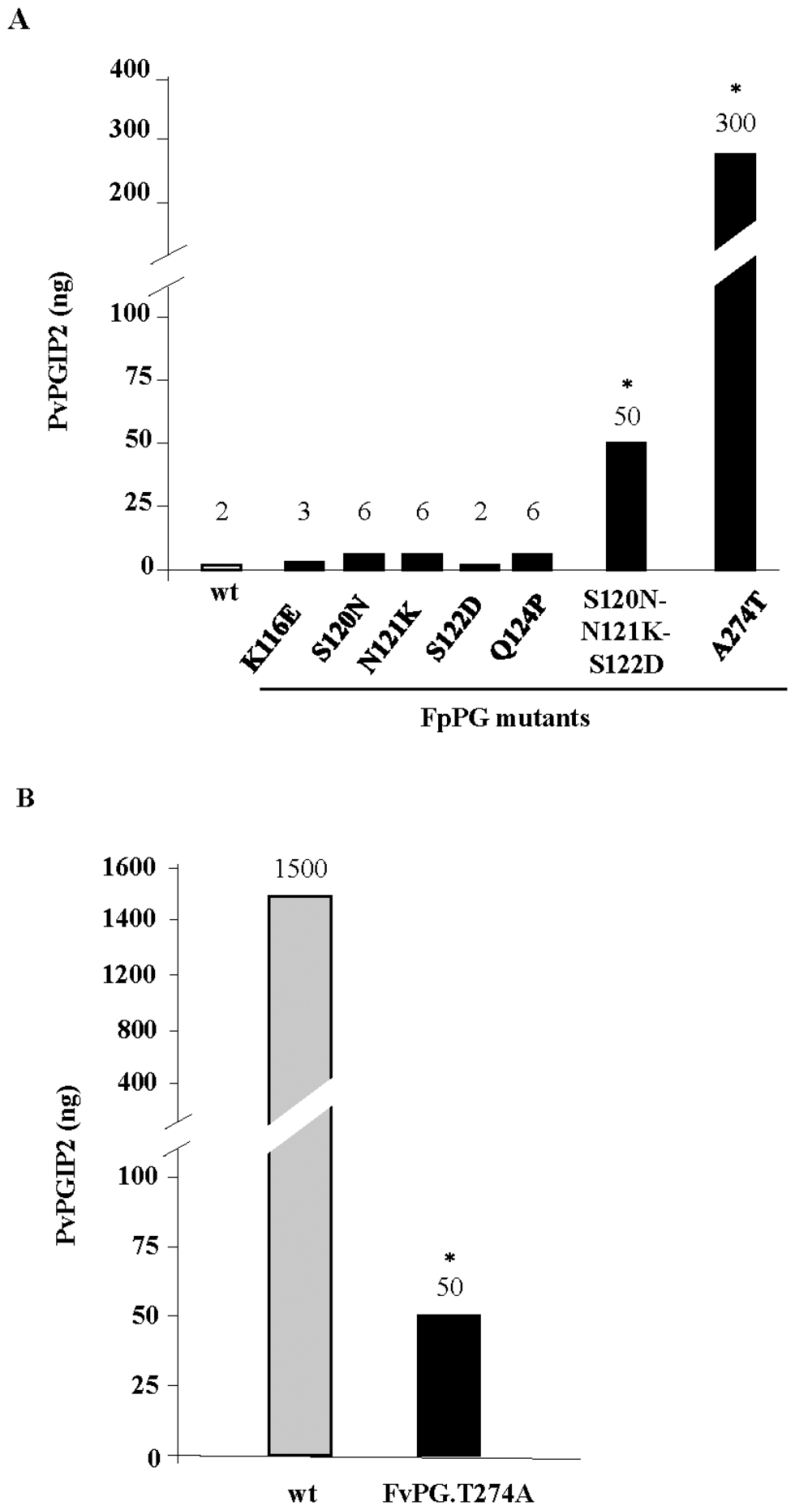
Inhibitory activities of PvPGIP2 against wild type (wt) and mutated forms of FpPG (A) and FvPG (B). The amount of PvPGIP2 (ng) causing 50% inhibition of one agarose diffusion unit of the indicated PGs at pH 4.7 are shown. Asterisks indicate statistically significant differences with wt PGs, according to Student's t test (*, P < 0.003).

Single variations at residues S120, N121, S122 and Q124 have only a modest effect on the capability of FpPG to be inhibited by PvPGIP2 (Figure 5A). Instead, the triple mutant FpPG.S120N-N121K-S122D is inhibited with a 25-fold reduced efficiency (Figure 5A). The FpPG.K116E variant, chosen as a negative control, was inhibited similarly to the wild-type (Figure 5A). Notably, the single mutation A274T, located in the proximity (^≈^8Å) of residue L303 at the C-terminal edge of the active site cleft, caused a marked loss of inhibition by PvPGIP2 (150-fold). These data suggest that the interacting area at the C-terminal edge of the active site cleft contributes to the majority of the binding energy in the PvPGIP2-FpPG complex formation, while the contribution of the interacting surface at the N-terminal edge of the active site is limited, albeit not negligible. 

The residue A274 of FpPG therefore appears to play a key role in the interaction; in agreement with this hypothesis, this position is occupied by an alanine both in FpPG and CluPG1 (Figure 3A). Therefore, in a gain-of-function approach, we generated the FvPG.T274A variant. Remarkably we show that this single mutation, which does not detectably alter enzymatic activity (Figure S1), enables the FvPG enzyme to be recognized by PvPGIP2 (Figure 5B).

## Discussion

The PG-PGIP interaction is a useful model system for analyzing the complex evolution of protein-protein recognition in plant-pathogen interaction for a number of reasons. First, the inhibitory spectra of PGIPs from different plant species or PGIP isoforms from the same plant towards PGs produced by several pathogens are remarkably different. For instance, PGs produced by *Aspergillus niger* (AnPG2), *Colletotrichum lupini* (CluPG1) and *Botrytis cinerea* (BcPG1) are inhibited by both isoforms PGIP1 and PGIP2 from *Phaseolus vulgaris*, as well as by PGIPs from other species [9,28,29,32]. Conversely, the PG produced by *Fusarium phyllophylum* (FpPG) is inhibited only by PvPGIP2 [9,29], while the closely related PG from *Fusarium verticilloides* (FvPG) is not inhibited by any PGIP so far characterized [68]. An additional complexity arises by investigating the kinetics of inhibition played by the prototypical PvPGIP2 inhibitor. Depending on the PG partner, the mechanism of inhibition may be competitive, non-competitive or mixed [31-33,59]. The complexity emerges also from the interaction analysis at the single residue level. A number of PvPGIP2 residues under selective pressure for diversification may contribute or not to the complex formation depending on the PG partner [27,69]. To rationalize these findings, we proposed that PvPGIP2 might inhibit PGs from different fungi by engaging them in complexes of different shapes and using diverse sets of interacting residues [9]. To fully validate this hypothesis, we previously determined the low resolution structure of the FpPG-PvPGIP2 complex showing that PvPGIP2 contacts the enzyme using the concave surface of its LRR solenoid in a head-to-head orientation [30]. In this work, we extend the SAXS analysis to the complex that PvPGIP2 establishes with CluPG1. This enzyme was chosen because is inhibited by PvPGIP2 with a non-competitive mechanism [33], unlike FpPG that is inhibited competitively by the same inhibitor [59]. The structure obtained for the CluPG1-PvPGIP2 complex shows that the inhibitor still uses the concave surface of its LRR solenoid to contact the enzyme, but the shape of the complex is markedly different. While CluPG1 is engaged only at the C-terminal edge of its active site cleft, FpPG is engaged at both the N- and C-terminal edges (see Figures 2B-2C and 3B-3C for comparison). Therefore, the active site cleft of FpPG is covered at both sides and this probably prevents the housing of the large polymeric substrate. On the contrary, the active site cleft of CluPG1 is still partially accessible to the substrate suggesting that the formation of a ternary enzyme-substrate-inhibitor complex is possible. These structural data fit with the different inhibition kinetics observed and provide a confirmation of our proposal, highlighting the versatility of PvPGIP2 in recognizing different PGs. 

FpPG contacts the inhibitor with residues belonging to a loop of sequence ^120^SNSNQ^124^, located at the N-terminal edge of the active site cleft. The corresponding loop in CluPG1 is shorter by one residue and is characterized by a different sequence (GGKT; see Figure 3A). This may explain why PvPGIP2 does not recognize this area of CluPG1. The second area recognized by PvPGIP2 is located at the C-terminal side of the cleft in both enzymes and is partially conserved both topologically and at the residue level. We replaced the FpPG L303, K310 and S363 residues with the corresponding ones of CluPG1 (see Table I and Figure 3A) and no difference in the inhibition capability played by PvPGIP2 was observed, as expected. However, analysis of the inhibition properties of the PvPGIP2.Q224K variant revealed that this variant does not recognize FpPG but inhibits the FpPG L303E variant. Therefore, while the Q224K replacement in PvPGIP2 abolishes a hot spot at the C-terminal side of the active site cleft of FpPG [30], the L303E mutation in the enzyme adds a residue, already present in CluPG1, that is critical for the interaction with PvPGIP2. Indeed, the L303 residue in the FpPG-PGIP2 complex faces several PvPGIP2 amino acids, such as Y107, T129, D131 and T155 and its replacement to glutamate may cause the formation a new hydrogen-bond interaction that is not possible with the wild-type leucine.

The combined analysis of the SAXS structures and mutational data enabled us to analyze the behavior of the FvPG enzyme that, despite being 92.5 % identical to FpPG, is not inhibited by PvPGIP2, as well as by any other known PGIP. We first demonstrated that the lack of inhibition is due to the PvPGIP2 failure to form a complex with FvPG (see Figure 4). Then we analyzed the differences at the two areas of FpPG that are recognized by PvPGIP2. The ^120^SNSNQ^124^ loop located at the N-terminal side of the active site cleft of FpPG is replaced in FvPG by a loop of sequence ^120^NKDNP^124^, with just one residue conserved out of five. Instead, the C-terminal interacting area is well conserved and just the A274T single amino-acidic variation is observed (see Figure 3A). We mutated residues of FpPG into the corresponding ones of FvPG. Single site mutations at the ^120^SNSNQ^124^ loop had only a modest effect on the inhibitory capability of PvPGIP2 (see Figure 5A). On the other hand, the mutation of FpPG A274 into the corresponding threonine of FvPG resulted in a remarkable 150-fold decrease of PvPGIP2 inhibition efficiency. Since the FpPG A274 residue faces F80 and Y105 in PvPGIP2 its mutation to threonine may add steric hindrance or cause a loss of hydrophobic stabilizing interactions. Interestingly, the PvPGIP2 Y105 residue was previously shown to be subjected to positive selection for the interaction [27].

These findings suggest that the residue at position 274 acts as a molecular switch that allows FvPG to escape recognition by PvPGIP2. To support this hypothesis, in a gain of function approach, we mutated the FvPG T274 into the corresponding alanine of FpPG and we observed, remarkably, that this single mutation is sufficient to confer to this enzyme the capability of being inhibited by PvPGIP2 (see Figure 5B).

In conclusion, in this work we investigated the interaction between PvPGIP2 and several PGs at the structural level and obtained evidences that enhance our understanding of this interaction. PvPGIP2 is a versatile inhibitor that is able to form complexes of different shapes with PGs from different pathogens. The inhibitor establishes complex interactions with the PG counterparts with several residues involved in the concave side of its LRR solenoid. On the enzyme side, residues in the proximity of the active site cleft are always recognized. However two different interaction areas may be identified, depending on the PG involved. Only when both of them are engaged the inhibition occurs with a competitive mechanism. The interaction area located at the C-terminal edge of the active site cleft is always recognized. It shows a higher degree of conservation and appears to play a major contribution when compared to the other interacting area. Within this area, just single site variations may change the fate of an interaction. This perfectly fits with an arms race scenario where PGs evolve to escape recognition by PGIPs while, on the opposite side, PGIPs evolve to enhance their inhibition spectra. We may exploit in the future the knowledge gained about the PG-PGIP interaction to develop *in vitro* PGIP variants that are able to inhibit PGs that are currently escaping recognition.

## Supporting Information

Table S1 **Primers used in this study.**
(PDF)

Table S2 **Summary of the low resolution structural data obtained from SAXS analysis of the CluPG1-PvPGIP2 complex.**
(PDF)

Figure S1 **Western blot analysis using a polyclonal antibody against the FpPG, on the mutated and wild-type forms of FpPG and FvPG producing an equal enzymatic activity on the agar diffusion assay.**
(PDF)
