# Supplementary material for: A Single Amino-Acid Substitution Allows Endo-Polygalacturonase of Fusarium verticillioides to Acquire Recognition by PGIP2 from Phaseolus vulgaris
Source: PLoS One. 2013 Nov 19;8(11):e80610. doi: 10.1371/journal.pone.0080610 (PMC3834070; doi:10.1371/journal.pone.0080610)
Supplement: Table S1 — Primers used in this study. (PDF) [file pone.0080610.s001.pdf]

Supplemental Table I. Primers used in this study.

Primers name	Sequence
**PGIP2.Q224K Fw**	CGGATCAGATAAGAACACGAAGAAGATACATCTGGCGAAG
**PGIP2.Q224K Rv**	CTTCGCCAGA TGTATCTTCTTCGTGTTCTT ATCTGATCCG
**PGIP2.Q224E Fw**	CGGATCAGATAAGAACACGGAGAAGATACATCTGGCGAAG
**PGIP2.Q224E Rv**	CTTCGCCAGA TGTATCTTCTCCGTGTTCTTATCTGATCCG
**FvPGEcoFw**	ACCTGAGAATTCGATCCCTGCTCCGTGAC
**FvPGXbaRv**	GCCTATCTAGACTAGCTGGGGCAAGTGTT
**FwFpK116E**	CAGGCGTACTGGGATGGCGAAGGTTCTAACAGCAATAGC
**RvFpK116E**	GCTATTGCTGTTAGAACCTTCGCCATCCCAGTACGCCTG
**FwFpS120N**	GGATGGCAAAGGTTCTAACAACAATAGCAACCAAAAGCCCG
**RvFpS120N**	CGGGCTTTTGGTTGCTATTGTTGTTAGAACCTTTGCCATCC
**FwFpN121K**	GGCAAAGGTTCTAACAGCAAGAGCAACCAAAAGCCCGATC
**RvFpN121K**	GATCGGGCTTTTGGTTGCTCTTGCTGTTAGAACCTTTGCC
**FwFpS122D**	GGCAAAGGTTCTAACAGCAATGACAACCAAAAGCCCGATCAC
**RvFpS122D**	GTGATCGGGCTTTTGGTTGTCATTGCTGTTAGAACCTTTGCC
**FwFpQ124P**	GTTCTAACAGCAATAGCAACCCAAAGCCCGATCACTTCATCG
**RvFpQ124P**	CGATGAAGTGATCGGGCTTTGGGTTGCTATTGCTGTTAGAAC
**FwFpA274T**	GCATCAAGTCCAACTCTGGCACAACTGGCACGATCAACAACG
**RvFpA274T**	CGTTGTTGATCGTGCCAGTTGTGCCAGAGTTGGACTTGATGC
**FwFpS120N-N121K**	GGGATGGCAAAGGTTCTAACAACAAGGACAACCAAAAGCCCGATCA C
**RvFpS120N-N121K**	GTGATCGGGCTTTTGGTTGTCCTTGTTGTTAGAACCTTTGCCATCCC
**FwFpL303E**	GTCGATGTTCAGCAGGACTATGAGAACGGCGGCCCTACTGGAAAG
**RvFpL303E**	CTTTCCAGTAGGGCCGCCGTTCTCATAGTCCTGCTGAACATCGAC
**FwFpK310T**	CGGCGGCCCTACTGGAACGCCGACCAACGGAGTCAA
**RvFpK310T**	TTGACTCCGTTGGTCGGCGTTCCAGTAGGGCCGCCG
**FwFpS363K**	GGTGGTGGCAAGACTAGCAAGTGCAACTATCCTACCAACACT
**RvFpS363K**	AGTGTTGGTAGGATAGTTGCACTTGCTAGTCTTGCCACCACC
**FwFvT274A**	GCATCAAGTCCAACTCTGGAGCAACTGGCACGATCAACAACG
**RvFvT274A**	CGTTGTTGATCGTGCCAGTTGCTCCAGAGTTGGACTTGATGC
**FwFvL303E**	GTCGATGTCCAGCAAGATTATGAGAACGGCGGTCCTACTGGAAAG
**RvFvL303E**	CTTTCCAGTAGGACCGCCGTTCTCATAATCTTGCTGGACATCGAC
