# Supplementary material for: A Single Amino-Acid Substitution Allows Endo-Polygalacturonase of Fusarium verticillioides to Acquire Recognition by PGIP2 from Phaseolus vulgaris
Source: PLoS One. 2013 Nov 19;8(11):e80610. doi: 10.1371/journal.pone.0080610 (PMC3834070; doi:10.1371/journal.pone.0080610)
Supplement: Table S2 — Summary of the low resolution structural data obtained from SAXS analysis of the CluPG1-PvPGIP2 complex. (PDF) [file pone.0080610.s002.pdf]

Supplemental Table II. Summary of the low resolution structural data obtained from SAXS analysis of the CluPG1-PvPGIP2 complex. Radius of gyration (R_g), maximum size of the particle (D_{max}), estimated volume, radius of gyration obtained with SASREF (R_{gS}), mean χ obtained with GA_STRUCT and SASREF, respectively (χ_G and χ_S) are reported. *Data for the FpPG-PvPGIP2 complex are taken from Reference 30 and are reported for comparison.

Sample	R_g (Å)	D_{max} (Å)	Volume (nm^3)	R_{gS} (Å)	$\langle\chi_G\rangle$	$\langle\chi_S\rangle$
CluPG1-PvPGIP2	28.5 ± 0.4	84 ± 2	109 ± 4	28.8	0.203 ± 0.009	0.49 ± 0.04
*FpPG-PvPGIP2	28.6 ± 0.4	80 ± 5	114 ± 6	28.3	0.416 ± 0.033	0.76 ± 0.02
