# Supplementary material for: A Single Amino-Acid Substitution Allows Endo-Polygalacturonase of Fusarium verticillioides to Acquire Recognition by PGIP2 from Phaseolus vulgaris
Source: PLoS One. 2013 Nov 19;8(11):e80610. doi: 10.1371/journal.pone.0080610 (PMC3834070; doi:10.1371/journal.pone.0080610)
Supplement: Figure S1 — Western blot analysis using a polyclonal antibody against the FpPG, on the mutated and wild-type forms of FpPG and FvPG producing an equal enzymatic activity on the agar diffusion assay. (PDF) [file pone.0080610.s003.pdf]

Supplemental Figure 1.

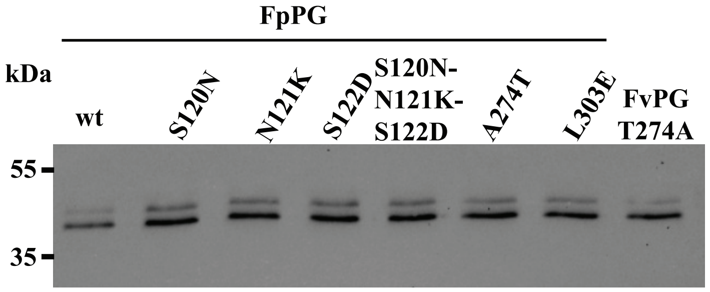

Supplemental Figure 1. Western blot analysis using a polyclonal antibody against the FpPG, on the mutated and wild-type forms of FpPG and FvPG producing an equal enzymatic activity on the agar diffusion assay.
